# Associations between Meteorological Factors and Reported Mumps Cases from 1999 to 2020 in Japan

**DOI:** 10.3390/epidemiologia2020013

**Published:** 2021-04-02

**Authors:** Keiji Mise, Ayako Sumi, Shintaro Takatsuka, Shin-ichi Toyoda

**Affiliations:** 1 Department of Admissions and High School Liaison, Center for Medical Education, Sapporo Medical University, Sapporo, Hokkaido 060-8556, Japan; mise@sapmed.ac.jp; 2 Division of Physics, Department of Liberal Arts and Sciences, Center for Medical Education, Sapporo Medical University, Sapporo, Hokkaido 060-8556, Japan; takatsuka@sapmed.ac.jp; 3 Department of Information Engineering, College of Industrial Technology, Amagasaki, Hyogo 661-0047, Japan; toyoda@cit.sangitan.ac.jp

**Keywords:** mumps, seasonality, surveillance, temperature, time series analysis

## Abstract

The present study investigated associations between epidemiological mumps patterns and meteorological factors in Japan. We used mumps surveillance data and meteorological data from all 47 prefectures of Japan from 1999 to 2020. A time-series analysis incorporating spectral analysis and the least-squares method was adopted. In all power spectral densities for the 47 prefectures, spectral lines were observed at frequency positions corresponding to 1-year and 6-month cycles. Optimum least-squares fitting (LSF) curves calculated with the 1-year and 6-month cycles explained the underlying variation in the mumps data. The LSF curves reproduced bimodal and unimodal cycles that are clearly observed in northern and southern Japan, respectively. In investigating factors associated with the seasonality of mumps epidemics, we defined the contribution ratios of a 1-year cycle (*Q*_1_) and 6-month cycle (*Q*_2_) as the contributions of amplitudes of 1-year and 6-month cycles, respectively, to the entire amplitude of the time series data. *Q*_1_ and *Q*_2_ were significantly correlated with annual mean temperature. The vaccine coverage rate of a measles–mumps–rubella vaccine might not have affected the 1-year and 6-month modes of the time series data. The results of the study suggest an association between mean temperature and mumps epidemics in Japan.

## 1. Introduction

Mumps is an acute respiratory disease caused by a virus belonging to the family *Paramyxoviridae*. Direct contact with mumps patients is regarded as the most common transmission route, because infectious air droplets can invade the upper respiratory tract mucous membrane [1]. Mumps is generally considered to be less contagious than measles or chickenpox, which may explain why so many children reach adulthood without having been immunized by naturally acquired infection [2]. The incubation period averages 18 days and ranges from 14 to 21 days [3]. Onset of the disease is characterized by inflammation of the parotid gland with precursory fever [1]. Although most infections are mild, severe clinical cases can occur involving complications such as aseptic meningitis, encephalitis, pancreatitis and orchitis. Mumps is one of the main causes of sensorineural deafness acquired during childhood, which is difficult to cure.

The treatment of mumps and its complications is basically symptomatic treatment. For fever, analgesics and antipyretics are administered, and for patients with meningitis, rest is instructed. Infusion is indicated in cases of dehydration. Vaccines are the only way to effectively prevent mumps [4]. Currently, 122 countries in the world implement the standard two-dose schedule as a routine vaccination [5]. Among developed countries, only Japan has not yet included the mumps vaccine in their routine vaccination schedule; this omission in Japan is due to a measles–mumps–rubella (MMR) vaccine being discontinued in April 1993 on account of the occurrence of aseptic meningitis following vaccination, which was attributed to the mumps vaccine component in the MMR vaccine [3]. Mumps vaccine coverage has ranged from 30% to 40% throughout Japan in recent years, which creates a level of herd immunity insufficient to prevent outbreaks, and large mumps epidemics recur at 4- to 5-year intervals [3]. Mumps remains a serious public health issue in Japan.

Seasonal distribution of mumps has been detected in various countries including Japan [6,7,8,9,10,11,12,13,14,15]. A seasonal pattern has been detected in the USA, with significant peaks in April [6]. The seasonal distribution reportedly varies between northern and southern China, where there are respective peaks in spring and summer [7], and an analysis of the weekly reported cases of mumps in Japan revealed that seasonal peaks were not identical from year to year in the southern part of the country [8]. Such distributions indicate that meteorological factors may influence the transmission of mumps. The effects of meteorological factors on mumps transmission may differ from one country to another, and they may differ within the same country in different climatic regions. To investigate the underlying causes of mumps epidemics in specific climatic regions, a systematic study is needed. Such an investigation should quantify the influence of meteorological factors on mumps incidence in different countries in each climatic region. Some studies have also investigated the effects of population density as a socio-economic factor on temporal variations in mumps epidemics in developing countries [16,17].

Previous studies investigating associations between epidemic patterns of reported mumps cases and meteorological conditions have focused on only one region each, such as Taiwan [14], or even multiple cities for one climatic zone each, such as Guangxi (southern China) in the subtropical monsoon region [16]. Meanwhile, Japan is divided into 47 prefectures, extending from latitude 45° N to 20° N, and its meteorological conditions thus vary widely; the most northern prefecture has a subpolar climate, and the most southern prefecture has a subtropical climate. Furthermore, the 47 prefectures have a range of population density from 71 to 5986 people/km^2^, which is wider than that (from 96 to 383 people/km^2^) for cities in Guangxi (southern China). As part of Japan’s nationwide infectious diseases reporting and surveillance system, mumps surveillance data have been collected in all 47 of the country’s prefectures since mid-1999. We surmised that a subset of the mumps surveillance data may be useful in clarifying associations of meteorological conditions and the density of the population experiencing a mumps epidemic. The present study conducted a time series analysis, incorporating the maximum entropy method (MEM) in a spectral analysis and the least-squares method (LSM) [18,19,20]. The effect of vaccinations on mumps epidemics was also investigated. The results obtained may facilitate the more accurate prediction of epidemics and more informed preparation for the effects of climatic changes on the epidemiology of infectious diseases.

## 2. Materials and Methods

### 2.1. Materials

The surveillance system of infectious diseases in Japan started to collect and publish weekly reported mumps incidence data for the whole of Japan in July 1981. Since April 1999, the incidence data have been published by all 47 prefectures of Japan. The incidence data indicate the number of mumps cases reported weekly per pediatric sentinel clinic. There are approximately 3000 pediatric sentinel clinics nationwide. Sentinel mumps cases were defined by clinical presentation; that is, sudden swelling of the parotid glands on one or both sides lasting longer than 2 days [21].

#### 2.1.1. Mumps Data by Prefecture of Japan from April 1999 to December 2020

To investigate the association between the reported number of mumps cases and meteorological conditions and population density in detail, a time series analysis was conducted for the longest possible weekly incidence data for the 47 prefectures in Japan currently available; i.e., from April 1999 to December 2020. The present study is the first to perform a time series analysis of the data for this period. The data were obtained from the Infectious Diseases Weekly Report Japan [21].

We selected three representative sites from the 47 prefectures in Japan: (a) Hokkaido Prefecture, the most northern (latitude 43° N); (b) Tokyo Prefecture, the capital city in the east (latitude 35° N); and (c) Okinawa Prefecture, the most southern (latitude 26° N). Hokkaido has a subpolar climate, Okinawa has a subtropical climate, and Tokyo has a temperate climate. In Table 1, the three prefectures are arranged from northern to southern Japan by latitude and longitude. The 47 prefectures of Japan were shown in our preceding study [18].

#### 2.1.2. Mumps Data for the Whole of Japan from July 1981 to December 2020

The effect of vaccination on mumps epidemics was investigated with the longest possible weekly incidence data of mumps for the whole of Japan currently available during the period July 1981 to December 2020. The present study is the first to perform a time series analysis of the data for this period. The data were obtained from the Surveillance of Infectious Disease [22] and the Infectious Diseases Weekly Report Japan [21]. The data are indicated in Dataset S1.

#### 2.1.3. Meteorological Data

In the present study, the daily mean temperature (°C), relative humidity (%), rainfall (mm), and wind velocity (m/s) were used, based on a study conducted in Japan’s southern prefecture reporting that mean temperature and relative humidity were associated with an increased occurrence of mumps [8]. These data were collected at stations that are part of the Automated Meteorological Data Acquisition System [23], which operates in Japan’s 47 prefectural capitals, and were obtained from the Japan Meteorological Agency website [24]. Daily data were obtained for a total of 7671 days from 1999 to 2020 (7671 data points). Using the daily data for mean temperature, relative humidity, and wind velocity from 1999 to 2020 for each prefecture we calculated a mean value corresponding to the average of the daily data (one data point). For each prefecture, we also calculated a summation of the daily rainfall from 1999 to 2020 (one data point).

### 2.2. Methods

#### 2.2.1. Time Series Analysis

We used a time series analysis consisting of MEM spectral analysis in the frequency domain and LSM in the time domain [18,19,20]. The MEM is considered to have a high degree of resolution of spectral estimates [25]. Therefore, an MEM spectral analysis allows us to determine precisely short data sequences, such as the infectious disease surveillance data used in this study [18,19,20,25,26,27].

##### MEM Spectral Analysis

We assumed that the time series data *x*(*t*) (where *t* is time) were composed of systematic and fluctuating parts [28]:

*x*(*t*) = systematic part + fluctuating part. (1)

To investigate temporal patterns of *x*(*t*) in the monthly time series data, we performed MEM spectral analysis [18]. This method of analysis facilitates elucidation of periodicities in a time series of short data lengths with a high degree of frequency resolution compared with other analysis methods of infectious disease surveillance data such as the fast Fourier transform and autoregressive methods, which require time series of long data lengths [29]. MEM spectral analysis produces a power spectral density (PSD).

The MEM-PSD *P*(*f*) (where *f* represents frequency) for a time series with equal sampling interval Δ*t*, can be expressed by

(2) $$P\left(f\right)=\frac{P_{m}\Delta t}{{\left|1+\sum_{k=-m}^{m}\gamma_{m.k}\exp\left[-i2\pi fk\Delta t\right]\right|}^{2}},$$

where the value of *P_m_* is the output power of a prediction-error filter of order *m* and *γ_m, k_* is the corresponding filter order.

##### LSM

The validity of the MEM spectral analysis results was confirmed by calculating the least squares fitting (LSF) curve pertaining to the original time series data *x*(*t*) with MEM-estimated periods. The formula used to generate the LSF curve for *X*(*t*) was as follows:

(3) $$X(t)=A_{0}+\sum_{n=1}^{N}A_{n}\cos\left\{2\pi f_{n}\left(t+\theta_{n}\right)\right\}.$$

The above formula is calculated using the LSM for *x*(*t*) with unknown parameters *f_n_*, *A*_0_ and *A*_n_ (*n* = 1, 2, 3, …, *N*), where *f_n_* (=1/*T_n_*; *T_n_* is the period) is the frequency of the *n*-th component; *A*_0_ is a constant that indicates the average value of the time-series data; *A_n_* is the amplitude of the *n*-th component and *θ_n_* is the phase of the *n*-th component; and *N* is the total number of components. The LSM using Equation (3) must be nonlinear. Linearization of this nonlinearity is required to obtain unique optimum values of these parameters. In the present analysis, linearization was achieved using the MEM-estimated periodic modes (*f_n_*). The value of *f_n_* can be determined by the positions of the peaks in the MEM-PSD. The optimum values of parameters *A*_0_, *A_n_* and *θ_n_* (*n* = 1, 2, 3, …, *N*) in Equation (3), except for *N*, were exactly determined from the optimum LSF curve (Equation (3)) calculated with *f_n_*. The reproducibility level of *x*(*t*) (Equation (1)) by the optimum LSF curve (Equation (3)) was evaluated via Spearman’s correlation (ρ) analysis performed using SPSS (Statistical Package for the Social Sciences) version 17.0J software (SPSS, Japan). A *p* value of ≤ 0.05 was considered statistically significant.

#### 2.2.2. Contribution Ratio

Based on the result of MEM spectral analysis, we assign periodic modes *f_n_* in Equation (3) that construct seasonal variations of mumps data. First, the power of each periodic mode is evaluated by the square of amplitude, *A_n_*^2^, of the *n*-th mode constituting the LSF curve *X*(*t*) (Equation (3)). Second, we estimate *R* corresponding to the power of residual time series which is obtained by subtracting the LSF curve *X*(*t*) (Equation (3)) from the original time series *x*(*t*) (Equation (1)). As a result, the total powers of the original time series *Q* is obtained by

(4) $$Q=A_{n}^{2}+R.$$

Dividing both sides of Equation (4) by *Q*, we obtain the normalized relation

(5) An2Q+RQ=1,

where *A_n_*^2^/*Q* and *R*/*Q* respectively correspond to the contribution of *A_n_*^2^ and *R* to, *Q*. We refer to the first term on the left-hand side of Equation (5) as the “contribution ratio”, which means the contribution *A_n_*^2^ normalized by *Q* [16,17,18]. If *A_n_*^2^/*Q* in the first term becomes large, then the second term *R*/*Q* becomes small. The formula used to generate the contribution ratio *Q_n_* is

(6) Qn=An2Q,

where *A_n_* indicates the amplitude of the *n*-th periodic mode constituting the LSF curve *X*(*t*) (Equation (3)) pertaining to the original data *x*(*t*) (Equation (1)), and *Q* is the total power of *x*(*t*).

#### 2.2.3. Segment Time Series Analysis

The effect of vaccinations on mumps epidemics was investigated adopting segment time series analysis, which has been widely used in fields such as medical and biological science, as well as in the physical sciences and engineering [30,31,32]. In segment time series analysis, weekly incidence data of mumps for the whole of Japan in the period July 1981 to December 2020 were divided into 200 segments. The segments each had a time range of 5 years and their starts differed in intervals of 2 months. The MEM-PSD was then calculated for each segment. The 200 MEM-PSDs thus obtained were arranged in the order of the time sequence to construct a three-dimensional (3D) spectral array.

#### 2.2.4. Outline of the Analysis Procedure

MEM spectral analysis was conducted first, and the long-term period was determined from the PSD for the time series data. Long-term trends in the data were then calculated using the LSF method (Equation (3)) with the MEM-estimated period. This LSF curve corresponding to the long-term trend was removed by subtracting the LSF curve from the data, and the residual time series data were thus obtained. The MEM-PSDs of the residual time were then calculated. The seasonality of mumps epidemics was investigated with contribution ratios (Equation (6)) for periodic modes of the residual data. Segment time series analysis was finally conducted.

## 3. Results

### 3.1. Number of Mumps Cases and Mean Daily Meteorological Data

From April 1999 to December 2020 a total of 2,315,511 cases of mumps were reported in Japan. The number of patients aged 3–6 years reportedly accounts for approximately 60% of the total number of mumps patients [3]. Descriptive statistics for the weekly meteorological data are shown in Table 2. The overall mean daily temperatures from 1999 to 2020 were 9.3 °C in Hokkaido (latitude 43° N), 16.6 °C in Tokyo (latitude 35° N), and 23.4 °C in Okinawa (latitude 26° N).

### 3.2. Temporal Variations in Mumps Incidence Data

**Figure 1:**
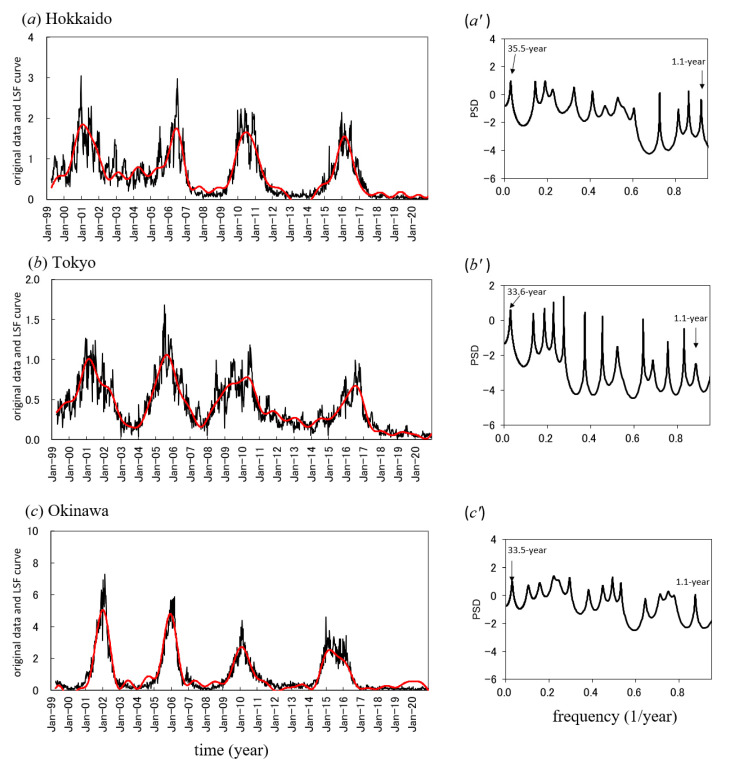
Weekly incidence data mumps in Japan from April 1999 to December 2020. (**a**–**c**) Comparison of the least squares fitting curves calculated for long-term trends (red line) in mumps data (solid line) for Hokkaido, Tokyo, and Okinawa. (**a’**–**c’**). Power spectral density in mumps data for Hokkaido, Tokyo, and Okinawa.

The three weekly incidence datasets gathered from April 1999 to December 2020 are shown in Figure 1. All incidence data exhibited long-term oscillations of an approximately 3- to 5-year period with shorter-term variations within a 1-year cycle. In Hokkaido (Figure 1a) and Tokyo (Figure 1b), the long-term oscillations were largely modulated by relatively irregular shorter-term variations within the long-term cycles. In Okinawa (Figure 1c), a long-term cycle was evident.

### 3.3. Long-Term Periodicities of the Mumps Incidence Data

The PSDs, P(*f*) (*f* [1/year]: frequency), were calculated for all the time series data shown in Figure 1a–c, and the respective results are shown in Figure 1a’–c’ (*f* ≤ 0.95). In each PSD, the most dominant spectral peak was observed during an approximately 3- to 5-year period, and the longest period appeared as a prominent peak at a frequency position longer than the length of the original data (20 years and 9 months, from April 1999 to December 2020)—for example, a 33-year period for Hokkaido (Figure 1a’). For the spectral peaks observed in the frequency range of the long-term periodic mode (>1 year), corresponding periods for the three prefectures are listed in Table 3. Using the periods listed in Table 3, the long-term trends in the mumps data for each prefecture were estimated via LSF using Equation (3). The results are shown in Figure 1a–c. The LSF curves for all prefectures reproduced the long-term trends in the original mumps data well. The good fit of the LSF curve to the original data is supported by the high respective ρ values of 0.91, 0.89 and 0.95 for Hokkaido, Tokyo, and Okinawa prefectures. Thus, the LSF curves are regarded as representative of the long-term variations in the original incidence data.

### 3.4. Short-Term Periodicities of the Mumps Incidence Data

**Figure 2:**
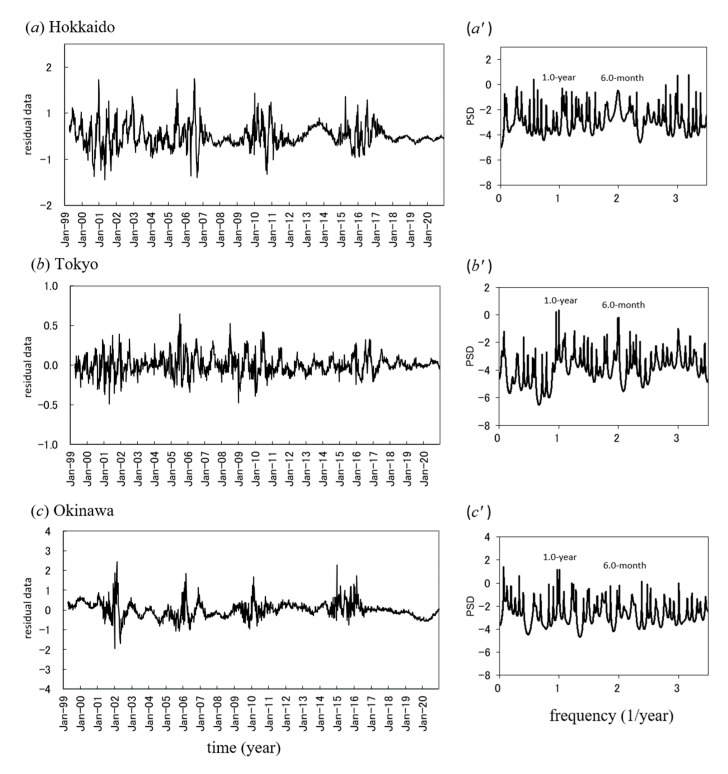
Seasonality of mumps incidence. (**a**–**c**) Residual time series data obtained by subtracting the long-term trends in mumps data from the mumps data for Hokkaido, Tokyo and Okinawa. (**a’**–**c’**) Power spectral density of the residual time series data for Hokkaido, Tokyo, and Okinawa.

The residual data obtained by subtracting the LSF curves from the original data are shown in Figure 2a–c. By using these residual data, periodicities in mumps data within periods of less than 1 year were investigated. The PSDs for the residual data are shown in Figure 2a’–c’. In each PSD, a prominent spectral peak was observed at *f* = 1.0 (=*f*_1_), corresponding to a 1.0-year period, and a spectral line of *f*_2_ = (*f*_1_ × 2) corresponding to the 6-month cycle was observed at *f* = 2.0. For each PSD (Figure 2a’–c’) the prominent spectral peak at *f*_2_ (6 months) is a point of interest because it evokes the question of whether the *f*_2_ mode has its origin in the harmonics of *f*_1_, in the 6-month cycle (bimodal cycle), or in a superposition of both.

### 3.5. Associations between Mumps Incidence and Meteorological Conditions and Population Density

**Figure 3:**
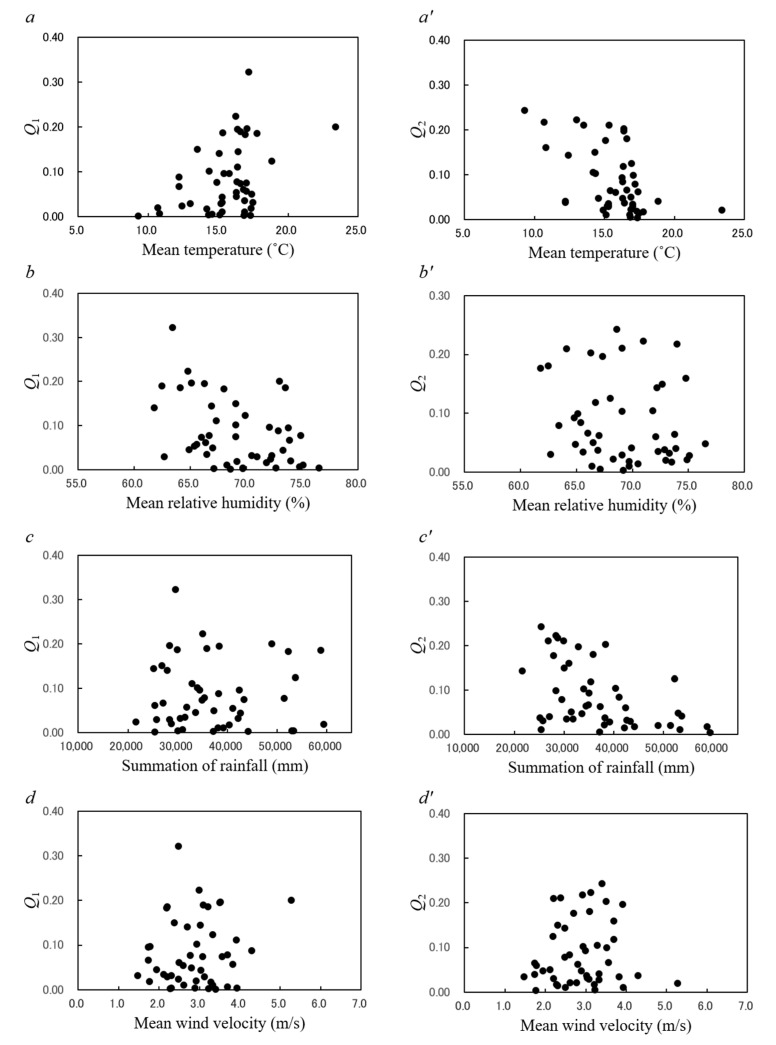
Plots of *Q*_1_ (left-hand side) and *Q*_2_ (right-hand side) against meteorological factors in 47 prefectures in Japan from 1999 to 2020. (**a**,**a’**) Daily mean temperature (°C). (**b**,**b’**) Daily relative humidity (%). (**c**,**c’**) Summation of daily rainfall (mm). (**d**,**d’**) Daily wind velocity (m/s).

**Figure 4:**
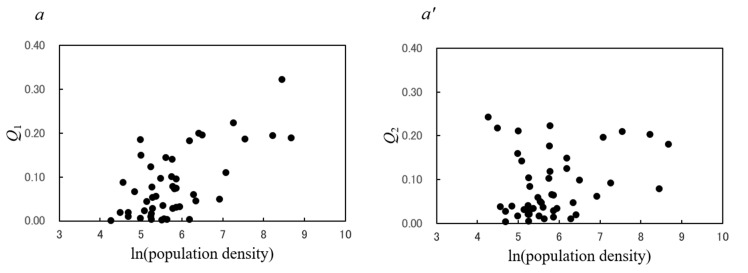
Plots of *Q*_1_ (**a**) and *Q*_2_ (**a’**) against population density in 47 prefectures in Japan from 1999 to 2020.

Figure 3 shows plots of the contribution ratios of the 1-year cycle (*Q*_1_; panels a–d) and the 6-month cycle (*Q*_2_; panels a’–d’) by mean temperature, relative humidity, rainfall, and wind velocity data for all 47 prefectures. Figure 4a,a’ show respective plots of *Q*_1_ and *Q*_2_ by population density for all 47 prefectures. Spearman’s ρ correlation coefficients between the contribution ratio (*Q*_1_ and *Q*_2_) and meteorological data and population density were calculated, and the results are shown in Table 4.

### 3.6. Unimodal Cycles in the Mumps Incidence Data

*Q*_1_ was significantly correlated with mean temperature (ρ = 0.331, *p* < 0.05; Figure 3a) and relative humidity (ρ = –0.381, *p* < 0.01; Figure 3b) but not with rainfall (ρ = –0.032, *p* = 0.832; Figure 3c) or wind velocity (ρ = 0.084, *p* = 0.573; Figure 3d). *Q*_1_ increased as the population density increased, although there was some scattering of points (ρ = 0.514, *p* < 0.01; Figure 4a). These results indicate that the unimodal cycle of reported cases of mumps in Japan is significantly associated with temperature, relative humidity, and population density.

### 3.7. Bimodal Cycles in the Mumps Incidence Data

*Q*_2_ was significantly correlated with mean temperature (ρ = –0.479, *p* < 0.001; Figure 3a’) and rainfall (ρ = –0.479, *p* < 0.01; Figure 3c’) but not with relative humidity (ρ = –0.203, *p* = 0.171; Figure 3b’), wind velocity (ρ = 0.078, *p* = 0.603; Figure 3d’), or population density (ρ = 0.182, *p* = 0.222; Figure 4a’). These results indicate that the bimodal cycle of reported cases of mumps in Japan is associated with the mean temperature and rainfall.

### 3.8. Peak Months of Mumps Epidemics

**Figure 5:**
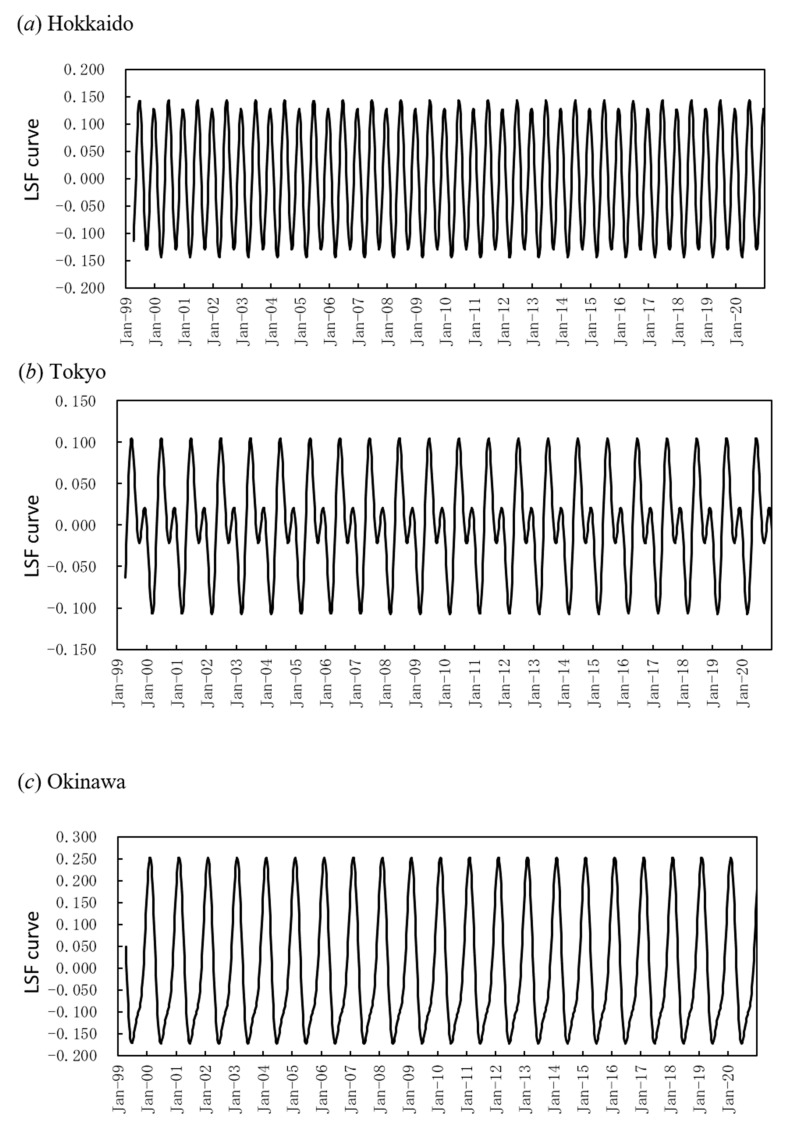
Least squares fitting curves calculated with 1-year and with 6-month periodic modes for the residual data. (**a**). Hokkaido, (**b**). Tokyo, and (**c**). Okinawa.

To investigate the peak months of mumps epidemics, the LSF curves for the residual data (Figure 2a–c) were calculated with the 1-year and the 6-month periodic modes. The results are shown in Figure 5a–c. The respective correlations between the residual data and the LSF curves in Figure 5a,b, and c were ρ = 0.36, 0.49, and 0.34. The peaks in the LSF curve for Hokkaido (Figure 5a) were in early summer (June) and winter (December). For Tokyo (Figure 5b) the peaks in the LSF curve were also in early summer (June) and winter (December). For Okinawa (Figure 5c) the peaks in the LSF curve were in winter (February).

### 3.9. Effect of Vaccination on Periodic Structures of Mumps Epidemics

**Figure 6:**
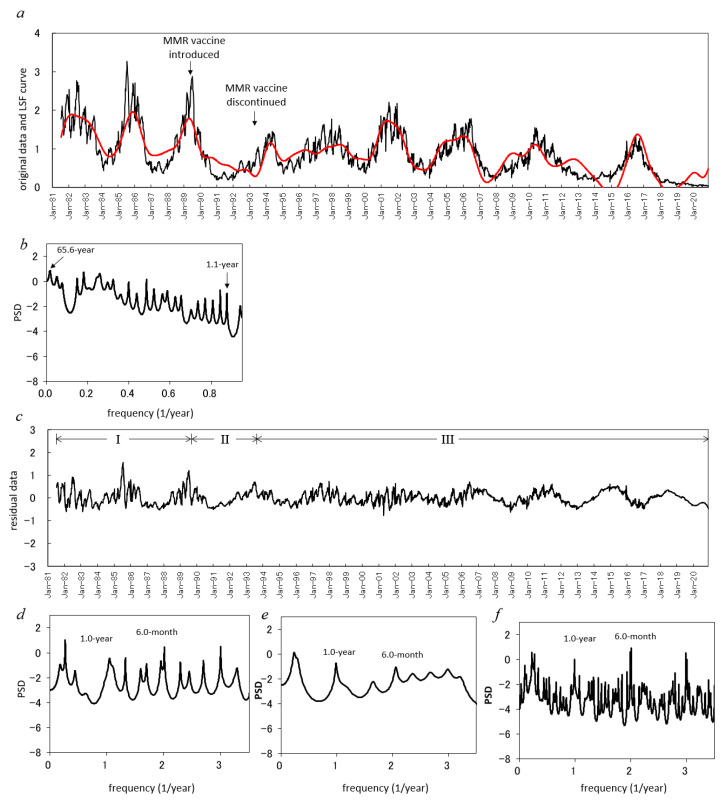
Effect of vaccination on periodicities of mumps epidemics. (**a**). Comparison of the least squares fitting curve calculated for the long-term trend (red line) in mumps data for the whole of Japan (solid line) from July 1981 to December 2020. (**b**). Power spectral density in mumps data. (**c**). Residual time series data. (**d**–**f**) Power spectral densities of the residual time series data for phases I, II, and III.

To quantitatively estimate the effect of mass vaccination on the 1-year cycle and 6-month cycle of mumps epidemics, we analyzed the incidence data of mumps for the whole of Japan during 1981–2020, as shown in Figure 6a. Therein, a decreasing trend of the incidence data was observed at the beginning of the MMR vaccine, which was started in April 1989 and discontinued in April 1993. The average incidence from July 1981 to March 1989, when mumps vaccination was completely voluntary, is approximately 1.33 (per 100,000). The average incidence from April 1989 to March 1993 when the MMR vaccination program was introduced and that from April 1993 to December 2020 when mumps vaccination was completely voluntary again are 0.73 and 0.72, respectively, and are reductions of 55% and 54%, respectively, as compared with the average incidence before the MMR vaccine was introduced (from July 1981 to March 1989).

The time-series analysis of the incidence data (Figure 6a) was conducted with the same procedure used for the prefecture’s data shown in Figure 1a–c. First, a spectral analysis of the original data (Figure 6a) was performed, and the PSD was obtained (Figure 6b). The long-term periods (>1 year) determined from the PSD (Figure 6b) are listed in Table 5. Next, the long-term trend was calculated as the LSF curve with Equation (2) (Figure 6a). This trend was removed by subtracting the LSF curve from the original data, and the residual data were obtained (Figure 6c).

The residual data (Figure 6c) were divided into three ranges (phases I, II, and III) in accordance with the starting and ending points of MMR vaccination (April 1989 and March 1993, respectively). The three ranges are labeled as phase I (July 1981 to March 1989), phase II (April 1989 to March 1993), and phase III (April 1993 to December 2020).

For the residual data in phases I, II and III (Figure 6c), MEM-PSDs were calculated. Semi-log plots of the PSDs are shown in Figure 6d,e, and f for phases I, II, and III, respectively. In each PSD (Figure 6d–f), common prominent peaks were observed at approximately ƒ = 1.0 and ƒ = 2.0, corresponding to the 1-year cycle and 6-month cycle of epidemics, respectively. *Q*_1_ values for phases I, II, and III are 0.13, 0.14 and 0.09, respectively. *Q*_2_ values for phases I, II and III are 0.24, 0.12, and 0.12, respectively.

**Figure 7:**
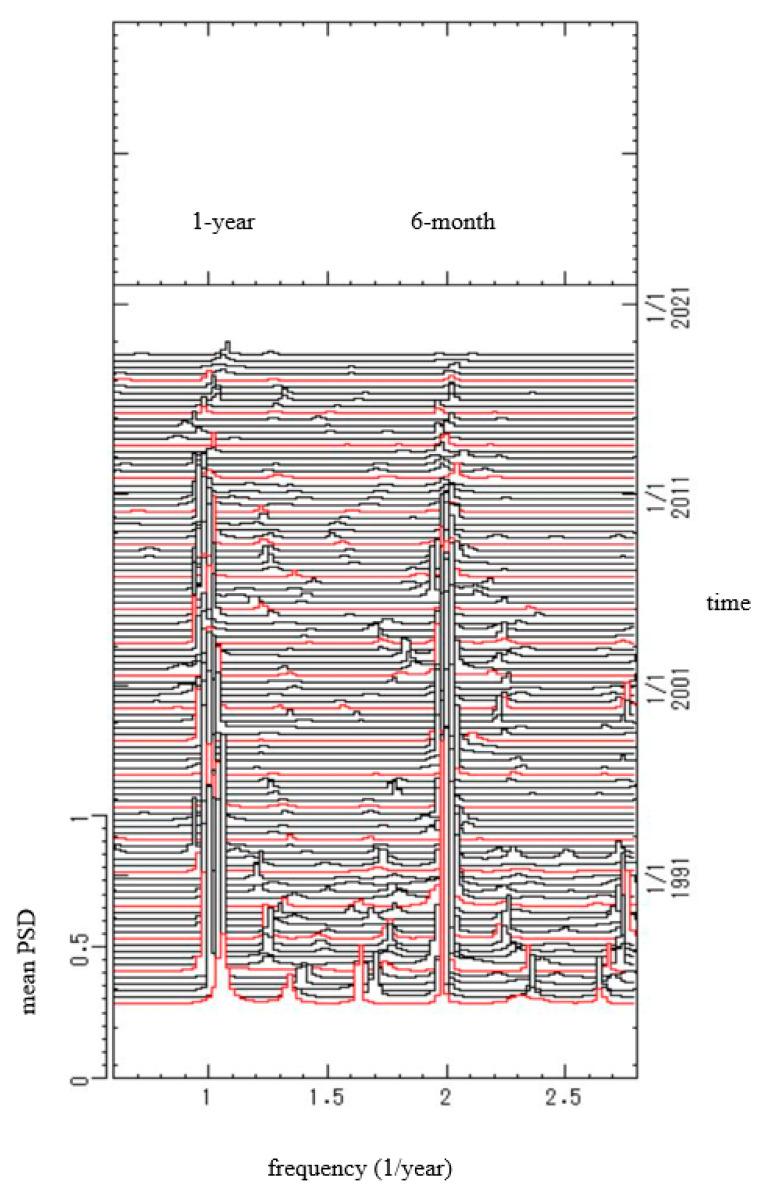
Three-dimensional spectral array for the residual data (Figure 6c).

To further investigate the effect of vaccination on periodic structures of mumps epidemics, segment analysis was conducted for the residual data (Figure 6c). All the residual data (Figure 6c) were divided into 200 segments. The segments each had a time range of 5 years, and the beginning of the range was delayed by 2 months. The PSD was then calculated for each segment. The 200 PSDs thus obtained were arranged in the order of the time sequence to construct the 3D spectral array, as shown in Figure 7, in which frequency is represented on the horizontal axis and time on the perpendicular axis running from bottom to top. In Figure 7, spectral peaks at the frequency *f* = 1.0 corresponding to a 1-year period and *f* = 2.0 corresponding to a 6-month period were unchangeably observed as a fine array over the entire time range.

## 4. Discussion

The present result that the occurrence of mumps was associated with the mean temperature and relative humidity was consistent with the results of previous studies conducted for Japan’s southern prefecture [8] and Taiwan [7]. With respect to the mean temperature, in the current study, there was a statistically significant relationship between the contribution ratio of the 1-year (*Q*_1_) and 6-month (*Q*_2_) cycles of reported cases of mumps and mean temperature (Figure 3a,a’). A similar relationship was observed with regard to reported cases of chickenpox in previous studies [20,33,34], and the observations are concordant with results reported by Shoji et al. [35]. Shoji et al. [35] showed that the incidence of chickenpox increased at temperatures of 5–20 °C (i.e., the temperature range at which the chickenpox virus is activated) and decreased at temperatures lower than 5 °C and higher than 20 °C. In regions of northern Japan, such as Hokkaido (latitude 43° N) where the temperature falls below 5 °C in winter and exceeds 20 °C in summer, the occurrence of chickenpox epidemics was bimodal [33]. In that same study bimodal cycles of chickenpox incidence were not evident at lower latitude, and unimodal cycles were evident in the southernmost prefecture, Okinawa (latitude 26° N), where the temperature rarely falls below 5 °C in winter and exceeds 20 °C in summer. This transition of patterns of chickenpox incidences in Japan was thought to depend on temperature [33]. With respect to mumps incidences, the present study found that the occurrence of epidemics transitions from bimodal cycles in Hokkaido (Figure 5a) to unimodal cycles in Okinawa (Figure 5c), as is the case for chickenpox. It is thus reasonable to hypothesize that temporal patterns of mumps incidence in Japan (Figure 5) are associated with temperature. This hypothesis is supported by the report that the mumps virus can tolerate environmental conditions remarkably well [36] and is relatively stable at 21 °C, and the reproduction of the mumps virus decreases when the external temperature is 4 °C and rapidly declines when the external temperature is 37 °C, resulting in a remarkable loss of infectivity [37,38].

*Q*_1_ and *Q*_2_, respectively, were significantly negatively associated with relative humidity (Figure 3b) and rainfall (Figure 3c’). The reasons behind the influence of relative humidity and rainfall on the transmission of mumps are unclear [15], but one potential explanation is that high relative humidity and large amounts of rainfall render outdoor activities unsuitable for children [11], which may in turn function to reduce the periodicity of mumps epidemics, resulting in the reduced *Q*_1_ associated with relative humidity (Figure 3b) and reduced *Q*_2_ associated with rainfall (Figure 3c’).

In Table 2, there is clearly large variance (corresponding to the value of SD/mean) in the daily rainfall data for the three prefectures. The amount of rainfall depends on the amount of water vapor in the atmosphere, which affects relative humidity [39]. The variance in the relative humidity for the three prefectures (Table 2) was relatively small compared with that for rainfall (Table 2). This finding is the result of relative humidity being constrained by the amount of saturated water vapor, which is dependent on air temperature [39]. It is thus reasonable to infer that unimodal and bimodal cycles observed in temporal variations of the reported mumps incidence were dominated by temperature.

We found no statistically significant association between wind velocity and *Q*_1_ (Figure 3d) and *Q*_2_ (Figure 3d’). Meanwhile, researchers found that the occurrence of mumps cases is positively associated with a wind speed of 1.8 m/s for Taiwan [14] and 2.2 m/s for Fujian province in southern China [12]. The mean values of the wind velocity of Fujian province (2.2 m/s) and Taiwan (1.8 m/s) are lower than those of 41 and 44 prefectures of all 47 prefectures in Japan, respectively. It is possible that there is a lower threshold effect below a wind speed of 2.2 or 1.8 m/s, which is not exceeded by the 41 and 47 prefectures in Japan, respectively.

The dominant summer peak relative to the winter peak observed in Tokyo (Figure 5b) may be associated with the observation that the degree of seasonality of mumps was significantly associated with population density (Figure 4a) and the fact that Tokyo has a much higher population density (5896 people/km^2^) than Hokkaido (71 people/km^2^) and Okinawa (605 people/km^2^). Given that it has been reported that patients aged 3 to 6 years account for approximately 60% of the total number of mumps cases [3], the present result that *Q*_1_ values of mumps varied with population density (Figure 4a) may be related to environmental and/or biological conditions affecting individuals aged 3–6 years. In that age group, there is a specific type of mumps infection risk. In Tokyo, people partake in outdoor activities more frequently in early summer, especially children, and this increases the likelihood of contact. This may cause the disease to spread more easily in Tokyo, which has a high population density, resulting in the dominant summer peak (Figure 5b).

From 1981 until recently, the vaccination coverage rate has remained low at approximately 30–40% [3], and the 1-year and 6-month modes are unchangeably observed as dominant spectral peaks in the PSDs for phases I, II, and III (Figure 6d–f, respectively) and in the 3D spectral array (Figure 7). Thus, the vaccination coverage rate might not have affected the 1-year and 6-month modes of the incidence data for the whole of Japan throughout the time range that was investigated in this study (1981–2020). When the vaccination coverage exceeds that required to prevent the spread of infection, 75–90% [3], the 1-year cycle and seasonal peak superposed on a 1-year cycle will diminish, as observed in Finland [40].

## 5. Conclusions

We confirmed that, in Japan, vaccination does not eliminate the seasonality of the mumps epidemics (Figure 6d–f and Figure 7). The control of mumps requires that the vaccination coverage exceeds that required to prevent the spread of infection (75–90%) [3] and, at the same time, the quantitative monitoring of the effect of the vaccination coverage on the 1-year and 6-month modes of the incidence data. The seasonality of the mumps epidemics has a significant correlation with meteorological factors (Figure 3), and we thus need to facilitate more informed preparation for the effects of climatic change on mumps epidemiology. We anticipate that the time series analysis methodology adopted in the present study, including MEM spectral analysis and LSM, will be useful in future studies investigating the seasonality of various medical conditions as well as mumps.

## Figures and Tables

**Table 1:** Longitude, latitude, population size and the peaks of least squares fitting curves derived for three prefectures in Japan.

	a. Latitude	b. Longitude	c. Population Size (×10^3^)	d. The Peak Month of LSF ^1^ Curve	
				First Peak	Second Peak
Hokkaido	43° N	141° E	5248	July	December
Tokyo	35° N	139° E	13,942	July	December
Okinawa	26° N	127° E	1454	February	---

^1^ least squares fitting.

**Table 2:** Mean, standard deviation and standard deviation/mean for the daily temperature (a), daily relative humidity (b), daily rainfall (c), summation of daily rainfall (d), and daily wind velocity (e) from 1999 to 2020 in three prefectures of Japan.

	a. Daily Temperature			b. Daily Relative Humidity			c. Daily Rainfall			d. Summation of Daily Rainfall (mm)	e. Daily Wind Velocity		
	Mean (°C)	SD ^a^ (°C)	SD/Mean	Mean (%)	SD ^a^ (%)	SD/Mean	Mean (mm)	SD ^a^ (mm)	SD/Mean		Mean (m/s)	SD ^a^ (m/s)	SD/Mean
Hokkaido	9.3	9.5	1.02	68.4	10.5	0.15	3.1	7.3	2.35	23414	3.1	7.3	2.35
Tokyo	16.6	7.9	0.48	62.1	15.2	0.24	4.3	13.2	3.07	32667	3.1	13.2	4.26
Okinawa	23.4	4.7	0.2	72.7	10.3	0.14	5.9	18.6	3.15	44198	5.3	18.6	3.51

^a^ standard deviation.

**Table 3:** Long-term periodic mode (>1 year) corresponding to the spectral peaks observed in the low-frequency range (*f* ≤ 1.1) of the power spectral densities (Figure 1a’–c’) for three prefectures in Japan.

Prefecture	Period (Year)
Hokkaido	35.5, 7.0, 5.3, 4.5, 3.1, 2.4, 2.1, 1.9, 1.7, 1.4, 1.2, 1.1
Tokyo	33.6, 7.4, 5.4, 4.4, 3.6, 2.7, 2.2, 1.9, 1.6, 1.5, 1.3, 1.2, 1.1
Okinawa	33.5, 9.6, 6.7, 4.5, 4.2, 3.4, 2.6, 2.2, 2.0, 1.9, 1.6, 1.4, 1.3, 1.1

**Table 4:** Spearman’s ρ calculated for population density, meteorological factors and contribution ratio of seasonal cycles (*Q*_1_ and *Q*_2_) for all 47 of Japan’s prefectures.

	*Q*_1_		*Q*_2_	
Temperature	0.331 *	(*p* < 0.05)	−0.479 **	(*p* < 0.01)
Relative humidity	−0.381 **	(*p* < 0.01)	−0.203	(*p* = 0.171)
Rainfall	−0.032	(*p* = 0.832)	−0.479 **	(*p* < 0.01)
Wind velocity	0.084	(*p* = 0.573)	0.078	(*p* = 0.603)
Population density	0.514 **	(*p* < 0.01)	0.182	(*p* = 0.222)

** *p* < 0.01, * *p* < 0.05.

**Table 5:** Long-term periodic mode (>1 year) corresponding to the spectral peaks observed in the low frequency range (*f* ≤ 0.95) of the power spectral densities (Figure 6b) for the incidence data of mumps for the whole of Japan from July 1981 to December 2020 (Figure 6a).

Period (Year)
65.6, 20.5, 13.4, 6.8, 5.6, 4.9, 3.9, 3.4, 3.1, 2.8, 2.5, 2.3, 2.1, 1.9, 1.8, 1.7, 1.6, 1.5, 1.4, 1.3, 1.2, 1.1

## Data Availability

The dataset of mumps analyzed during the current study are contained in Supplementary materials (Dataset S1). The data are also available from refs. [21,22].

## Supplementary Materials

The following are available online at https://www.mdpi.com/article/10.3390/epidemiologia2020013/s1, Dataset S1: Time-series data of the weekly incidence data from all 47 prefectures of Japan.
